# 'Single molecule': theory and experiments, an introduction

**DOI:** 10.1186/1477-3155-11-S1-S1

**Published:** 2013-12-10

**Authors:** Daniel Riveline

**Affiliations:** 1 Laboratory of Cell Physics, ISIS/IGBMC, Université de Strasbourg and CNRS (UMR 7006), 8 allée Gaspard Monge, 67083 Strasbourg, France; 2 Development and Stem Cells Program, IGBMC, CNRS (UMR 7104), INSERM (U964), Université de Strasbourg, 1 rue Laurent Fries, BP10142, 67400 Illkirch, France

## Abstract

At scales below micrometers, Brownian motion dictates most of the behaviors. The simple observation of a colloid is striking: a permanent and random motion is seen, whereas inertial forces play a negligible role. This Physics, where velocity is proportional to force, has opened new horizons in biology. The random feature is challenged in living systems where some proteins - molecular motors - have a directed motion whereas their passive behaviors of colloid should lead to a Brownian motion. Individual proteins, polymers of living matter such as DNA, RNA, actin or microtubules, molecular motors, all these objects can be viewed as chains of colloids. They are submitted to shocks from molecules of the solvent. Shapes taken by these biopolymers or dynamics imposed by motors can be measured and modeled from single molecules to their collective effects. Thanks to the development of experimental methods such as optical tweezers, Atomic Force Microscope (AFM), micropipettes, and quantitative fluorescence (such as Förster Resonance Energy Transfer, FRET), it is possible to manipulate these individual biomolecules in an unprecedented manner: experiments allow to probe the validity of models; and a new Physics has thereby emerged with original biological insights. Theories based on statistical mechanics are needed to explain behaviors of these systems. When force-extension curves of these molecules are extracted, the curves need to be fitted with models that predict the deformation of free objects or submitted to a force. When velocity of motors is altered, a quantitative analysis is required to explain the motions of individual molecules under external forces. This lecture will give some elements of introduction to the lectures of the session 'Nanophysics for Molecular Biology'.

## Introduction

A word of a caution about the style adopted in this review. The goal is not to present a formal lecture. The idea is rather to give intuitions about an experimental manner of understanding the systems beyond the formalism. This introduction is intended to be understood by students and scientists from a variety of backgrounds in Biology, Physics or Chemistry. If this approach is not academic, it may have the merit to give intuitive insights into the experimental visions of the living matter.

I start by giving basic ideas and estimates for the Physics associated with single molecules; I continue by presenting simple ideas in the Physics of single polymers. I conclude with basic concepts for molecular motors. Throughout the text, I refer explicitly to the elements useful for understanding the works presented in this session. More detailed reviews can be found in the following references [1-4].

### Brownian motion: elements for understanding single molecule experiments

**Figure 1:**
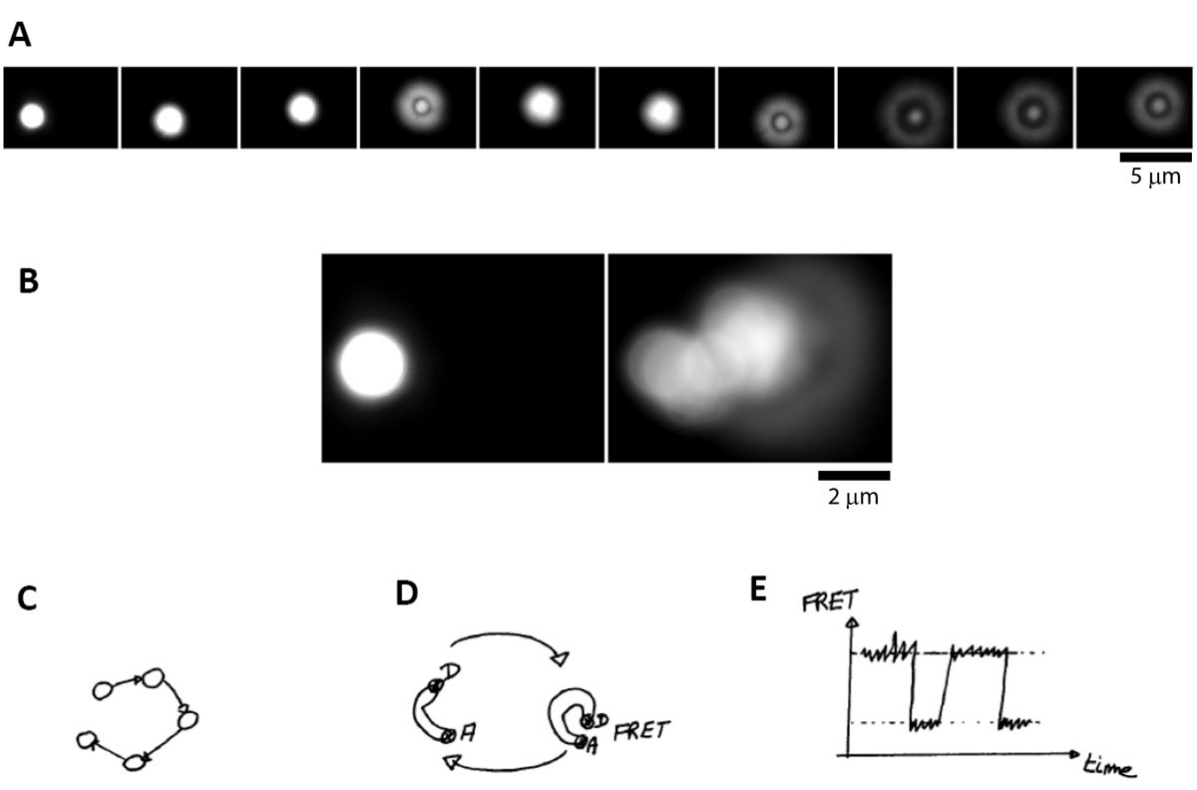
**Brownian motion and FRET**. (A) Brownian motion of a latex particle, 2 μm in diameter; time between frames 4 s; the movement is in 3-dimensions; in particular, panels 4 and 7-10 show that the bead does not remain on the same focal plane; the apparent shape of the bead provides the measure for the motion along the z-axis; (B) Starting point (left) and superposition of images over 44 s; (C) Schematics of the Brownian motion of a particle; (D) A flexible molecule experiences the same type of fluctuations, which can be revealed by FRET between a donor (D) and acceptor (A) pair of fluorophores; (E) The corresponding signal of fluorescence is shown as a function of time.

Brownian motion is well known. It is formally associated with the concept of entropy. But its basic and intuitive understanding is difficult. The observation of single colloids with an optical microscope is informative and striking: a 2 μm latex particle does undergo constant motion in water within seconds in the three dimensions (see Figure 1 and Additional File 1). The same experiment can be repeated with single colloids of different sizes and densities. These motions will be always observed - with different timescales and amplitudes. Apparently, beyond the chemical nature of the colloids, a conserved phenomenon is at play where sizes of the objects have a key role. In particular, the same type of motion is observed for colloids of 1 nm in diameter as well. This length corresponds to the size of single molecules: biomolecules such as DNA, RNA, proteins should therefore experience also this type of fluctuations.

### Orders of magnitude

This phenomenon has been described remarkably in the seminal paper 'Life at low Reynolds number' by Purcell [5]. Here we propose to estimate several values for parameters such as molecular forces, coefficient of diffusion. These *scaling *arguments are very common in Physics and they allow to reliably predict if an experiment is doable or correct, *a priori *and *a posteriori*.

Let's consider the colloid of a typical length a, its energy is k_B_T, where k_B _is the Boltzmann constant, and T the temperature. A natural scale for the force is set by:

(1) $$F\propto \frac{k_{B}T}{a}$$

We take the following values for numerical applications: a = 4 nm, k_B_T = 4 × 10^-21 ^J at room temperature. A force of 10^-12 ^N is expected, *ie *picoNewton forces. This simple estimate is remarkably close to measured values.

Values for the coefficient of diffusion D can also be estimated. We consider the Einstein relation given by:

(2) $$D=\frac{k_{B}T}{6\pi \eta a}$$

where η is the viscosity of the solvent. Relation (2) gives D as the ratio of the energy associated with Brownian motion over the friction with the solvent. We take η = 10^-3 ^Pa.s for water, and we obtain typically 5 μm^2^/ms for the estimate, again consistent with measurements performed with various techniques. It is interesting to note that the typical distance performed by a free protein will be of the order of $\sqrt{Dt}$: 10 μm in about 10 ms, which corresponds to the typical cell diameter. This motion will be anywhere in the cell though, in contrast to molecular motors that will transport proteins/lipids to specific locations within the cell in a targeted manner.

Beyond these estimates, the nature of this random motion is not intuitive. One would be tempted to say that it is associated with inertia. The observation of motion in everyday's life at the macroscopic level probably triggers this impression. When a ball is being kicked in a soccer game, the ball continues its trajectory when it has left the foot. In the same manner, the colloid would undergo a sustained motion because of the shocks provided by the molecules of the solvent.

As a way to determine whether this inertia is also at play at the scale of the colloid, a simple dimensionless parameter can be calculated, the *Reynolds number*. It is written as:

(3) $$\text{Re}\;=\frac{inertial\text{\_}forces}{viscous\text{\_}forces}=\frac{\rho av}{\eta }$$

where ρ is the density of water, a is the length of the colloid, and η the viscosity of water, and v the speed associated to typical forces

(4) $$v=\frac{F}{6\pi \eta a}$$

with F≈1 pN.

For water, ρ is about 10^3 ^kg/m^3^, η is about 10^-3 ^Pa.s, and we take again the typical length *a *of 4 nm.

The numerical application for the Reynolds number gives:

(5) $$\text{Re}\sim 10^{-4}<<1$$

So the viscous forces are much larger than the forces associated with inertia (see Eq. 3). In effect, the colloids are moving but after the shocks from the molecules of the solvent, they are very shortly stopped; the motion proceeds in a given direction or in another, because other shocks from other molecules arise.

This low Reynolds number shows that viscous forces *dominate *at this scale. Returning to the soccer ball image, this result suggests that the particle receives a kick but it immediately stops its motion. This non-intuitive behavior leads to a new way of viewing the systems; permanent motion and no inertia.

The consequences of this framework at the scale of single molecules are important: instead of having acceleration compensated by forces, *velocities *are equilibrated by *forces*. In other words, a force has always to be applied on single objects to trigger its motion.

### Fluctuations of polymers: microtubules, actin, DNA, proteins

**Figure 2:**
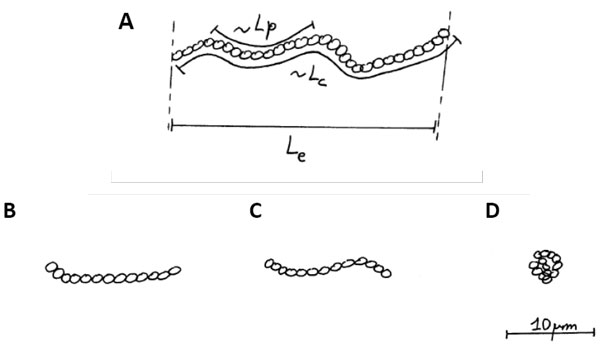
**The Worm-Like-Chain model**. (A) A polymer is viewed as a chain of colloids; Lp, persistence length, Lc, contour lengths, Le, end-to-end distance. Different polymers of the same contour length can be rigid (B), semi-flexible (C), or flexible (D); it is the ratio of Lc over Lp that determines the flexibility.

A polymer can be viewed in different manners. The atomic details are certainly playing a key role. They are essential in determining the specificity of interactions between different molecules for example. As presented by Patrick Schultz, the structure of the transcription factor can allow to reveal the mechanism of actions of transcription. But there is another way to describe these polymers: they can be viewed as a chain of colloids (see Figure 2). If k_B_T could move single particles as we said before, k_B_T can also promote some shocks between the medium and the polymer. Locally at the scale of the colloid within the chain of colloids, a deformation is promoted. But its longitudinal extent is limited by the neighbouring colloids within the chain. A local bent is appearing; it has a finite lifetime, other molecules of the solvent apply forces along the opposite transverse directions as well. These events occur at low Reynolds number again: there is no inertia associated with these motions. Such fluctuations of shapes have been observed for a variety of single polymers such as DNA, microtubules, actin filaments. It is important to note that typical biological polymers are nanometer thick, much below the size set by the resolution in optical microscopy, 200 nm; but their labelling by fluorescent probes allow to observe them in dynamics with fluorescent microscopy and their fluctuations can be captured with CCD cameras.

An intuition of rigidity or flexibility appears by just observing sequences of fluctuating polymers (see Figure 2B-D for schematics of three polymers of the same contour length). If the polymer remains straight, it would be said to be rigid; if it is exhibiting many undulations, a polymer gives the impression to be flexible. A natural parameter can then be identified, the *persistence length *Lp. Qualitatively, this length sets the scale along which the polymer keeps a deformation promoted by k_B_T. After this length, the polymer relaxes, and a new bending can occur. The polymer is viewed as a *Worm-Like Chain *(WLC). Typical values for persistence lengths are 5 mm for microtubules, 10 μm for actin filaments, 50 nm for DNA. Their respective aspects under the microscope are shown in Figure 2B, C, D for a 10 μm long polymer. Because the persistence length is a built-in dimension, a polymer cannot be said to be in essence rigid or flexible. It is *the ratio *between its contour length Lc and its persistence length Lp that sets the rule: if Lc>>Lp, the polymer is flexible; if Lc<<Lp, the polymer is rigid; if Lc~Lp, the polymer is said to be semi-flexible.

There are several methods to measure this persistence length Lp. One way consists in measuring the auto-correlation function of tangents along the polymer. The relation is given by:

(6) $$\left\langle t\left(s\right)\cdot t\left(0\right)\right\rangle =\left\langle \cos\theta \left(s\right)\right\rangle =e^{-\frac{s}{Lp}}$$

where **t**(s) is the unit tangent vector at the curvilinear position s on the polymer. By taking time lapse movies of fluctuations, this autocorrelation function can be plotted: the fit with Equation 6 allows to measure the persistence length Lp.

### Structure of biological macromolecules

Biological molecules have several levels of organisation. Proteins are made of sequences of amino acids: they assemble into *domains*. Strikingly these domains form entities that can assemble like *Lego *parts of living matter. Proteins with different localisations and functions in cells of various organisms can have similar domains. These homologies are instrumental guides to anticipate and probe molecular interactions.

These domains can have binding *sites *that allow the recognition of two interacting proteins. This *Lego* is powerful: a protein with domains will be represented schematically with parts, each part having a function such as binding *site *to a proteic partner or hydrolysis of Adenosine Tri-Phosphate (ATP) for example. The lecture of Patrick Schultz will illustrate how the proteins structures can reveal the mode of action of an enzyme [7].

Another level of organisation is important in cells. When the same protein or base can polymerize, we obtain filaments with high order. They often have a polarity. For example, actin filaments form helical structures with a pitch of 72 nm. Actin monomers are periodically organized typically every 5 nm. DNA is a double helix of a pitch of about 3 nm. Bases are periodically localized 0.3 nm along the chain. This larger order of organization provides long range correlation in cells.

Structures are important for unraveling the binding partners, organizations of domains. They are complemented by *dynamics *studies which give times and/or frequencies of search and interactions between macromolecules.

### Single molecules/Ensemble of molecules

Single molecules can fluctuate. With the vision of macromolecules as connected domains with flexible linkers, it is easy to see that shocks by the particles of the solvent can trigger the motion of domains and thereby probe the deformation of proteins.

When collection of biomolecules are placed in an experimental chamber, they fluctuate asynchronously. The overall signal is averaged out over all fluctuations. Single molecules have then to be studied individually. New methods have been designed to measure the dynamics of their fluctuations.

This type of approach is illustrated by the lecture of Ben Schuler. The energy provided by k_B_T is allowing to probe the folding of proteins. Shocks experienced by proteins are triggering deformations. Single proteins are fluctuating in shapes, and some domains are more flexible than others. How to reveal these deformations? The fluorescent approach *Förster Resonance Energy Transfer *(FRET, see Figure 1D-E) is a powerful way to do so. This method relies on dipole-dipole interactions between the electronic states of two fluorophores: these are specifically bound on two domains of single proteins, a donor D and an acceptor A. The molecular biology approaches have been shown to be powerful to succeed in these labelling at the single molecule level. When the donor is excited with light, it may trigger by resonance the excitation of the acceptor fluorophore if this latter is located in the vicinity, few nm typically. Depending on the proximities between the donor and the acceptor associated to their locations on the protein and its fluctuations triggered by k_B_T, the intensity of fluorescence is changing in a predictable manner with resolutions within nanometer. The efficiency of transfer, E, is given by:

(7) $$E=\frac{1}{1+{\left(\frac{R}{R_{0}}\right)}^{6}}$$

where R is the effective distance between the donor and acceptor, and R_0 _is the Förster radius, which corresponds to 50% transfer, typically around 1-10 nm.

The same experiment can be reproduced for donor and acceptor pairs placed on other locations of single proteins and the same experiment can be repeated. Therefore this FRET read-out provides a powerful method for characterising the folding of single proteins for example [6].

### Why measuring forces?

If structure allows to identify the binding sites between molecules, forces allow to reveal the energy involved in these interactions. Qualitatively, by considering an object in everyday's life, it is not possible to evaluate its resistance to deformation by its simple observation. Forces applied to single molecules allow the evaluation of binding interactions between domains in single proteins or between motors and their partners. Their dynamics of opening or their rules for stabilities or assembly and disassembly can therefore be predicted in a quantitative manner.

### Methods for manipulating single molecules

There are several methods that allow to measure forces on single molecules: pipettes, optical tweezers, magnetic tweezers [8], Atomic Force Microscopes (AFM). In the present school, Félix Rico reports experiments with AFM [9]. Similarities and differences between the different instruments can be found in [10]. Here I give the general principle guiding these measurements.

**Figure 3:**
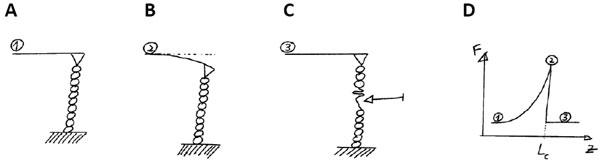
**Force spectroscopy of a single polymer by Atomic Force Microscopy**. (A) The polymer ends are grafted to a surface and to the cantilever; (B) the surface is lowered thereby causing the bending of the cantilever; (C) A domain (shown by the arrow) is opening and the cantilever returns to its original position; (D) The corresponding force-extension curve is shown.

The main read-out is the *extension *of a single molecule as a function of *force*. This curve gives the elastic signature of a molecule in a specific and unique manner. For a spring, this relation is linear, and the slope gives the spring constant. For single molecules, this elastic signature is non-linear. A typical example is shown in Figure 3 with AFM: a molecule is stretched between an AFM cantilever and a surface (Figure 3A); it elongates and the AFM tip is bending (Figure 3B), a domain is opened (Figure 3C); the elastic signature follows the generic force-extension curve of a Worm-Like Chain (between points 1 and 2 in Figure 3D). Its analytical expression is given by:

(8) $$\frac{FL_{p}}{k_{B}T}=\frac{x}{L_{c}}-\frac{1}{4}+\frac{1}{4}{\left(1-\frac{x}{L_{c}}\right)}^{-2}$$

where F is the force, x the extension, Lc and Lp the contour length and persistence length respectively.

It is worth noting that Equation (8) exhibits different regimes: an entropic linear regime where x<<Lc, the AFM tip unwrap the folded polymer ((1) in Figure 3D); the k_B_T Brownian motion is promoting its folding, and the force comes in opposition to this conformation 'disorder'; when x~Lc, the behaviour is quadratic: the domain is deformed ((2) in Figure 3D). For x = Lc, the analytical expression diverges ((3) in Figure 3D). This corresponds to the breakage of a domain. The fitting procedure consists of extracting this newly available length for single events of the domain opening, *ie *the parameter Lc in Equation (8).

Other types of force-extension curves have been reported with plateaux or non-linearities. A close comparison between their shapes and the associated fits allows to derive organisations and dynamics of biomolecules and supramolecular organelles such as chromatins, transcription machineries for example.

In order to estimate the proper extension promoted by the apparatus, single molecules need to be grafted by their proper ends. The Chemistry associated to the specific binding of nucleotides or residues has expanded significantly since the pioneering experiments performed in the late '90s. Two molecular glues are classically used: biotin-streptavidin, where the biotin group is specifically coupled to a nucleotide or a domain, and the Digoxigenin-anti-Digoxigenin, where the Digoxigenin is also specifically targeted on single molecules. It is important to note that these experimental steps are usually difficult and they require many tests and errors: they rely on a perfect association of molecular glues on every single molecule to be probed mechanically. Another difficulty of these experiments is associated to the success in pulling single molecules with optical tweezers, AFM, or magnetic tweezers. Tips, beads and proteins/DNA are incubated in an experimental chamber and the interactions will be occurring in a random manner. Usually, the experimentalist knows *a posteriori *that single molecules properly end-grafted were stretched by carefully observing the signature of the elastic curves; in addition, force-extension curves look different if single or multiple molecules are extended.

Single molecule experiments are difficult, but they have yielded unprecedented results in Biology. For example the folding of proteins were unravelled, the opening of domains in DNA and proteins were demonstrated, or the rules for the assembly of chromatin and chromosomes among many examples.

### Measuring pico-Newton forces and nanometer deformations

Equation (8) requires to measure two parameters, force and extension. The principle of the force measurement is based on the deformation of a spring of calibrated spring constant: by its deformation, the force can be inferred like in a weighing machine. If the design differs from apparatus to apparatus, the method is similar. For example, in optical tweezers, a bead is trapped with a focused laser: if the bead goes away from the center of the trap, there is a restoring force that promotes its return to its original position; this corresponds to a virtual spring that is experiencing an extension. In AFM, a cantilever is bent when a force is applied; the associated vertical spring constant allows to estimate forces when the deformation is measured (see Figure 3B). Calibration methods for the spring constants k are established: for low spring constants, the cantilever or the trapped bead undergo the thermally-driven Brownian motion introduced above. Its typical average amplitude is given by the equipartition theorem as a function of the temperature T and the effective spring constant k:

(9) $$\frac{1}{2}k\left\langle \Delta x^{2}\right\rangle =\frac{1}{2}k_{B}T$$

For larger spring constants, a needle of calibrated spring constant is deforming the spring probe to be characterised: the spring constant is extracted. Typical spring constants values range from pN/μm to nN/μm: displacements/deformations are nanometers in amplitudes for picoNewton forces in these experiments. Accordingly, cantilevers or laser beam powers are designed in such a way that the typical effective spring constants are spanning the pN/μm-nN/μm range.

In addition to calibrating the force sensors, there is a need to measure deformations at the nanometer scale. Usually, a laser beam is sent on the bead or on the cantilever. The beam is then reflected on a photodiode; changes in the beam position can be translated into forces. The extension of the molecule is measured simultaneously. As a result, force-extension curves are computed.

There are many ways to apply and measure forces: the application can be static and/or dynamics. If we compare the typical timescales and lengthscales of the protein fluctuations with the timescales and lengthscales of the force, we can anticipate some non-trivial effects in the force measurements; the *loading rate *parameter measures the variation of force with time; for a given spring constant, it is corresponding to the pulling speed. If the loading rate is large, the detachment force for single domains will be high; for infinitely slow loading rate, the detachment will be almost at zero force. An intuitive way of understanding this phenomenon is to pull on a *Post-it* paper attached on a table: a rapid pull will cause a sudden detachment, whereas a slow pull will lead to a slow detachment with low force.

These considerations have been probed and energy landscapes have been derived for several single molecules. If the latter measurements are certainly demanding experimentally, they allow to estimate more specifically the interaction potentials within molecules.

In conclusion, the manipulation of single molecules has allowed to unravel molecular mechanisms that were not accessible so far. The corresponding dynamics can also be studied in a powerful manner in these new approaches with single molecules and this feature is illustrated in the contributions of this session.

### Molecular motors: experiments and theory

**Figure 4:**
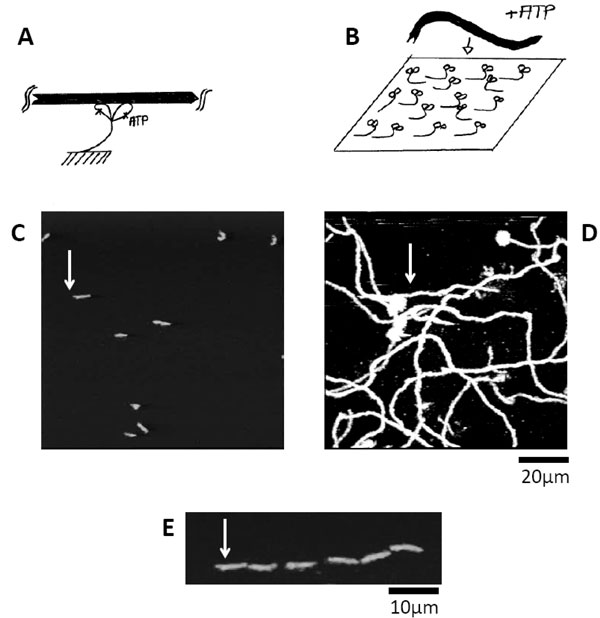
**Motility assays experiments with molecular motors**. (A) Myosin is shown with two globular heads, which can hydrolyze ATP; it binds actin filament (black filament); (B) In a motility assay experiment, myosin molecules are grafted on a surface; in the presence of ATP, actin filaments bind to the surface (C); They move directionally as shown by the superposition of succeeding images (D) or snapshots of the same filament over time (see (E), time between snapshots 5 s); the white arrow (C-D-E) shows the same filament.

As we said before, colloids or single molecules undergo a random motion. This motion can be rectified, *i.e*. with a source of energy usually associated with the hydrolysis of ATP/GTP, a molecule can move directionally along a track. The principle of a molecular motor - and its conceptual problem - was envisioned by Richard Feynman in one of his lectures [11]. This field of molecular motor has expanded in a remarkable manner since the '90s. In particular, the cell uses a variety of motors, ranging from force appliers and cargo transporters with wide ranges of biological functions in transcription, replication, transport, energy production, etc. Whenever an enzyme is undergoing a directional motion, it reveals a molecular motor behavior. A typical example is shown in Figure 4: myosin molecules are grafted on surfaces (Figure 4A and Figure 4B), actin polymers are fluorescently labelled (se Figure 4C), and their directed motions can be visualised by fluorescent microscopy (see Figure 4D, Figure 4E, and Additional File 2).

How can such a directed motion occur in an environment dominated by random Brownian motion at k_B_T? First, energy is required: it is provided by the hydrolysis of ATP; it can be shown that its amplitude is of the order of 10k_B_T. Then, a sustained motion in a given direction requires some specific features at low Reynolds number. When an actin filament encounters the surface, it binds to the motors and it keeps moving along the same direction. Apparently, it is the actin *filament *which imposes the direction of motion; the continuous motion could not occur otherwise. As I emphasized above, this structural asymmetry feature is established by actin filaments which are composed of two parallel double helices, as well as for many tracks associated to motors, such as microtubule, DNA, RNA for example. In addition, it is a *periodic *asymmetry, since the same myosin head will encounter subsequent portions of filaments (see Figure 4B).

However this asymmetry alone does not set the direction of motors associated with a given filament: some molecular motors move on one direction along a track, whereas other motors will move in the opposite direction, for example kinesins and dyneins on microtubules. It is the *interaction *between a given motor and its track with a broken symmetry that will impose the direction. To illustrate these ideas, let's recapitulate a cycle of interaction, by following a single myosin head grafted on the surface and bound to the actin filament: the myosin hydrolyses ATP, the motor gains energy, it then detaches and binds again to the filament on another monomer, while giving a kick always along the same direction set by its interaction with the polar filament. The filament moves; a new ATP binds the myosin head, ATP is hydrolysed, and the cycle starts again.

Altogether, three features are essential to explain the directed motion (also called *rectification*): energy of 10k_B_T, periodicity, broken symmetry of filaments/cables. These elements help to understand that myosin heads are 'pushing' the actin filament which imposes motion through its polarity.

In addition, trajectories exhibit two interesting features (Figure 4D): (i) they look like the crawling of worms, *i.e*. the motion of the tip of the filament is followed by the rest of the filament; and (ii) motions are not straight. These phenomena can be explained easily: the filaments are attached throughout their lengths to myosin heads; the tip is free to fluctuate to find a next myosin head to interact with; it may be located along the direction and the tip moves straight while myosin heads are pushing the track, the filament exhibits a motion looking like a crawling worm. But the fluctuations of k_B_T and the random deposition of heads on the surface may allow the tip to look for another head. As a result, the filament takes new turns.

Orders of magnitude associated with molecular motors can be estimated with simple arguments. As we reported above, the hydrolysis of ATP is of the order of 10k_B_T; the binding energy of the motor to the filament is also of the order of 10k_B_T - allowing the couple motor-track to 'resist' to Brownian motion with a lower energy, k_B_T. When the myosin has an elementary step in one direction, its amplitude a is known to be of the order of 1 nm. These scales of energy and length allow to set the typical force F associated with single motors. As stated before in Equation (1), we have the following scaling relation for the force F per motor:

(10) $$F\propto \frac{10k_{B}T}{a}$$

We obtain a force per molecular motor in the range of 10 pN, if we take a = 4 nm as in the case of Brownian motion. These forces have been measured by different methods (AFM, optical and magnetic tweezers for example), and they remarkably all give these ranges of forces.

The velocity of filaments can also be estimated: during a cycle of ATP hydrolysis, the myosin is bound for a fraction t_B _of the cycle time to the actin filament, typically 1 ms at 25°C. Since the elementary change of conformation *a *is of the order of 1 nm, the velocity v is of the order of:

(11) $$v_{B}\propto \frac{a}{t_{B}}$$

We obtain velocities of 1 μm/s which corresponds to the typical speeds in motility assays (see Figure 4 and Additional File 2). Single myosin heads velocity sets the velocity of the whole filament in a reliable manner.

Motors have several functions, and one parameter allows to have an intuition of their roles: the *duty ratio *r. If t_c _is the total time of one hydrolysis cycle of ATP, a fraction of this time t_B _is associated to the motor binding to its track. The duty ratio is set by

(12) $$r\propto \frac{t_{B}}{t_{C}}$$

If *r *is of the order of 90-100%, the motor is said to be *processive*. It spends most of its time on the track: its function is to transport material such as vesicles without losing its 'road' when k_B_T of the Brownian motion is challenging the interaction. In contrast, when r if of the order of 10% or below, the motor is most of the time unbound from the polymer; the motors have to act in concert to prevent the detachment from the track. However this dynamics is also allowing continuous motion: a short kick is given by myosin heads; if it would be too long, other myosin heads could be bound on the same filament in a non-synchronous manner and they could stall the filament; this would prevent motion;a short duty ratio therefore allows another head to give a kick subsequently without interfering with the kick of the former head. These *non-processive *motors are found in situations when cells apply forces, like in muscles or in stress fibers. This classification appears to be enlightening for a variety of motors.

Altogether key parameters can be estimated with simple scaling arguments, forces, velocities. Several models were developed for understanding quantitatively the dynamics of single motors and collection of motors. They take into account, the breaking of symmetry, the scale set by 10k_B_T, the time of the ATP/GTP cycles and the time of the bound state. They can lead to a variety of behaviours ranging from directed motion to collective effects such as oscillations.

**Figure 5:**
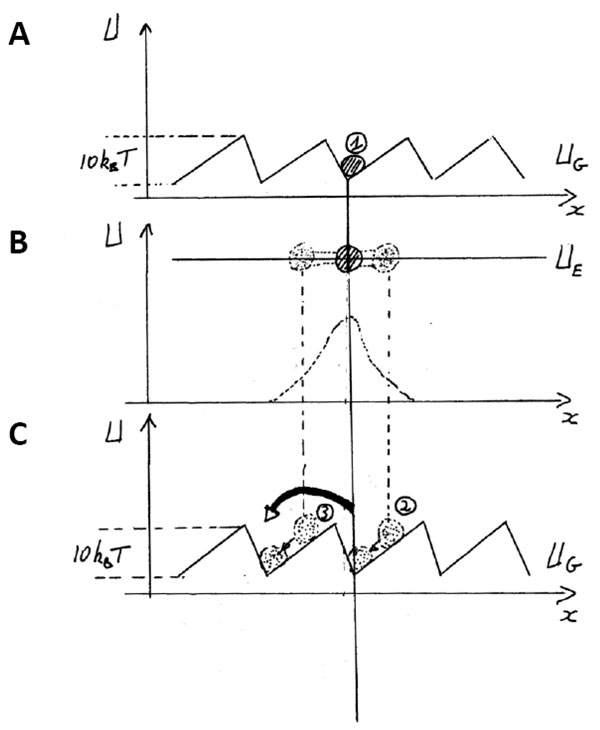
**Modeling molecular motors, the two-state model**. Interaction potentials as a function of space; (A) The particle is trapped in the minimum of interaction potential (1). When the particle is in the excited state, it undergoes a non-restricted Brownian motion and it diffuses (B); the distribution of positions is shown. When the ground state is applied again (C), more particles go to the left (3) than to the right (2). U_G_: interaction potential of the ground state; U_E_: interaction potential of the excited state.

Here we briefly detail such a model using interaction asymmetric potentials (see Figure 5, see also [12]). It allows to recapitulate and to integrate ideas from Brownian motion and from molecular motors. A motor can be viewed as a particle. Along the periodic structure of the polymer track, it binds on a site which minimizes its energy: the particle is localized in the minima of the interaction potential (Figure 5A). The height of the potential is about 10k_B_T, set by the energy scale of the motors. This interaction potential is asymmetric and periodic, which corresponds to two key features of tracks, as presented before.

When the particle is excited into the excited state (Figure 5B), it can diffuse with its k_B_T energy on a flat energy landscape. Subsequently, the ground potential is imposed again on the particle, the particle will find again the spatial minimum of energy (Figure 5C). But on average, some particles will have travelled more in one direction than in another, because the potential is asymmetric. As a result, the motion is rectified. It is worth noting that the ATP hydrolysis can be viewed as the *clock *corresponding to transition rates between both states.

In this description with interaction potentials and particles, the emphasis is *not *on the molecular details but on the physical laws defining the rules for rectifying motion. The problem can be written in equations and the phase diagram exhibit rich behaviours in terms of efficiency of rectification and dependence on energy profiles. The model has been extended to collective effects of motors, where many motors can cooperate when they interact with the same track: motors can oscillate collectively. This dynamic phase transition was demonstrated *in vitro *in motility assays and *in vivo *in developing embryos (*Drosophila *for example). Such approaches have suggested that collective effects can be critically important for morphogenesis [12].

## Conclusions

Assembly of molecules in test tubes usually hinder the behaviors of single molecules. The ensemble averages out the behavior of single molecules. Fluctuations are screened out, and this hinders the observation of molecular events. As a result, experiments on single molecules have proven their strengths in determining the dynamic properties of a variety of objects: free fluctuations with k_B_T (Ben Schuler lecture), elasticity measurements with tweezers or AFM (Félix Rico lecture).

A promising direction could be coming from experiments combining molecular biology, structure, and experiments on single molecules. Many remarkable results have recently been going along this line for example in Bustamante Laboratory [13]. They should allow to bridge the functions for each domain of proteins with the global dynamics when they act as single molecules in action.

Experiments with single molecules have revealed new features in Physics, in Chemistry and in Biology in the last 20 years. New intuitions need to be developed for experimentalists, new models need to be designed by theorists, paradigms are shifted for biologists from ensemble responses to single molecules fluctuations, from molecular explanations to emerging collective effects. Undoubtedly, future collaborations between these three fields will allow to unravel new phenomena relevant for understanding the behaviours of single molecules with unprecedented biological significance.

## List of abbreviations

DNA, RNA, AFM, FRET, CCD, WLC, ATP, GTP

## Competing interests

The author declares no competing interests.

## Supplementary Material

Additional file 1 **Movie 1 - Brownian motion of a colloid (2 μm in diameter)**. This movie shows the free 3D motion of a latex bead observed by fluorescence microscopy. Total time: 44 s. Click here for file

Additional file 2 **Movie 2 - Motility assay experiment with acto-myosin**. This movie shows the directed motion of actin filaments on a surface coated with myosin heads; the energy source ATP is in the medium. A typical filament is 10 μm long. Total time: 15 s. Click here for file
